# Discovery and validation of a multi-protein panel for predicting non-fatal major adverse cardiovascular events in diabetic kidney disease

**DOI:** 10.3389/fendo.2026.1883523

**Published:** 2026-08-27

**Authors:** Li Jiang, Chieh Chien, Tianli Li, Haojun Zhang, Tingting Zhao, Xiai Wu

**Affiliations:** 1 Diabetes Department of integrated Chinese and Western medicine; China National Center for Integrated Traditional Chinese and Western Medicine, China-Japan Friendship Hospital, Beijing, China; 2 Department of Internal Medicine, Mental Health Center of Dongcheng District, Beijing, China; 3 National Integrated Traditional and Western Medicine Center for Cardiovascular Disease, China-Japan Friendship Hospital, Beijing, China; 4 Beijing Key Lab for Immune-Mediated Inflammatory Diseases, Institute of Clinical Medical Sciences, China-Japan Friendship Hospital, Beijing, China

**Keywords:** diabetic kidney disease (DKD), machine learning, major adverse cardiovascular events (MACE), plasma proteomics, predictive model

## Abstract

**Objective:**

To identify plasma protein biomarkers associated with incident non-fatal major adverse cardiovascular events (MACE) in diabetic kidney disease (DKD) patients.

**Research design and methods:**

We analyzed 317 DKD patients from the UK Biobank. Plasma proteomics and clinical data (demographics, metabolism, renal function) were integrated. In an exploratory discovery phase, three sequential Cox regression models (crude, socio-demographic-adjusted, socio-demographic-metabolic adjusted) screened non-fatal MACE-associated proteins. To prevent information leakage, the cohort was then randomly split into training (70%) and testing (30%) sets; machine-learning feature selection, hyperparameter optimization, and final model development were performed exclusively within the training set. The associated proteins were input into the four-step machine-learning pipeline (LASSO-Cox, random survival forest, Boruta, XGBoost-Cox). Predictive performance was validated using Kaplan-Meier survival analyses, longitudinal trajectory modeling, and ROC benchmarking. An interactive web application was deployed for clinical implementation.

**Results:**

Of 1,463 plasma proteins, 561 were associated with non-fatal MACE across Cox models, with 14 overlapping proteins. Nine core proteins (ANG, IL1R1, CXCL14, ESAM, PTGDS, HAVCR1, FGFR2, IGSF8, CCL3) were validated: ANG showed the strongest non-fatal MACE association (HR = 3.88, 95%CI 2.33-6.48, p<0.001), and all high-expression groups had elevated non-fatal MACE risk. GO/KEGG enrichment highlighted inflammatory-immune pathways like positive regulation of MAPK cascade, Cytokine-cytokine receptor interaction and PI3K-Akt signaling pathway as key mechanisms. The model integrating proteins, demographic factors, and clinical variables achieved the highest predictive performance across non-fatal MACE (AUC = 0.768), myocardial infarction (MI) (0.808), and stroke (0.816) outcomes, with superior stability in cross-validation. CoxBoost + Elastic Net framework was selected as the optimal framework via benchmarking of 101 algorithms. The model demonstrated favorable calibration in high-risk patients and yielded positive net clinical benefit across decision thresholds of 5% to 45%. The web tool (https://jiangli2941.github.io/MACE-prediction-v2/) enables input of 28 variables, outputs non-fatal MACE risk status, risk probability, and highlights abnormal indicators.

**Conclusion:**

Plasma proteomics combined with machine learning identifies robust non-fatal MACE predictors in DKD.

## Introduction

Diabetic kidney disease (DKD) is an increasingly severe public health issue worldwide, with rising incidence and prevalence. Patients with DKD not only face the risk of kidney disease progression but also frequently suffer from major adverse cardiovascular events (MACE) such as myocardial infarction and stroke, placing a heavy burden on society and families (1). Although DKD itself follows a protracted and potentially modifiable course, the combination of microvascular basement-membrane thickening, hyper-coagulability, low-grade inflammation, and sustained renin–angiotensin–aldosterone system (RAAS) activation creates a vulnerable milieu in which the myocardium and cerebral vasculature predispose to ischaemia (2, 3). Consequently, life expectancy declines markedly after MACE, underscoring the imperative for early risk detection (4).

Contemporary advances in high-sensitivity troponins, NT-proBNP, and haemorheological biomarkers have refined our understanding of MACE pathophysiology (5). However, these indices typically rise only 6–12h after overt ischaemia, limiting their utility for primordial prevention (6). Structural imaging, such as cardiac perfusion scintigraphy or cerebral MRI, can depict metabolic derangements before symptoms emerge, but the associated costs, throughput constraints, and radiation exposure render these modalities impractical for large-scale screening (7, 8). There is therefore an urgent unmet need for an inexpensive, scalable tool that can anticipate MACE before irreversible organ damage occurs in the DKD population.

In recent years, research on blood-based biomarkers has made significant progress, especially in plasma proteomics (9, 10). In the context of cardiovascular and metabolic diseases (CMDs), these molecules have proven invaluable for mechanistic dissection and risk stratification (11). In this study, we leveraged the UK Biobank cohort of DKD participants to identify plasma proteins robustly associated with incident non-fatal MACE. We subsequently fused proteomic and clinical features within an ensemble machine-learning framework, and applied SHapley Additive exPlanations (SHAP) to render each prediction transparent. The resulting model not only stratified individuals at high future risk but also quantified the contribution of every variable, offering an interpretable, cost-effective strategy for early intervention and resource allocation in diabetic kidney disease.

## Methods

### Study population

The UK Biobank is a prospective, population-based cohort comprising over 500,000 individuals aged 39–70 years, recruited from 22 assessment centers across the United Kingdom between 2006 and 2010. This resource is linked to national death, hospitalization, and cancer registries and has released extensive data, including genome-wide genotypes, multimodal imaging, and high-throughput plasma proteomics (measured using the Olink^®^ Explore 3072 proximity-extension assay) for a probability sub-sample. We combined the previous similar studies (12) and the clinical guidelines (13) for diabetic kidney disease (DKD) to formulate the following inclusion criteria: Participants whose records included any ICD-10 code indicating diabetes with renal complications: E10.2, E11.2, E14.2, N08.3, or N18. Participants with an ICD-9 assignment of 250.3 (diabetes mellitus with renal manifestations).Among individuals already diagnosed with diabetes, those who (1) Received any CKD-specific ICD-10 code (N18.0-N18.9) or a CKD-related OPCS-4 procedure code (M01, X40), (2) Self-reported at baseline chronic kidney disease, renal failure, dialysis, or kidney transplantation, (3) Exhibited an estimated GFR < 60 ml/min/1.73 m² and/or a urinary albumin-to-creatinine ratio ≥ 30 mg/g for at least 3 months (checking between instance 0, 1 and 2), in accordance with the KDIGO guidelines. If repeated measurements were unavailable, estimated glomerular filtration rate (eGFR) from a single measurement was used. Diabetes was defined as followed: (1) Self-reported or diagnosed with diabetes by a doctor, (2) Currently taking antidiabetic medications or using insulin, (3) Patients with a glycated hemoglobin (HbA1c) level greater than 48 mmol/mol, or (4) Patients with a random venous blood glucose level greater than 11.1 mmol/L. The detailed codes in the UK Biobank were shown in **Supplementary Table 1**. The end of follow-up was defined as the latest available linkage date for hospital episode statistics, death registries, and primary care records, which for this analysis was September 2021 for participants in England. The median follow-up time for the study population was 10.2 years (interquartile range: 8.2-12.1 years).

### Plasma proteomics

Baseline blood was drawn at 22 assessment centers between 2007 and 2010. After EDTA anticoagulation, tubes were centrifuged at 2500 g for 10 min at 4 °C; plasma aliquots were stored at –80 °C and shipped on dry ice to the Olink core facility in Sweden. High-sensitivity proximity-extension immunoassays were used to quantify proteins in 54306 participants. The protein quantification was normalized according to the general method (14). Detailed protein definitions are provided in **Supplementary Table 2**. The Olink Explore 3072 proximity-extension assay (PEA) generates Normalized Protein eXpression (NPX) values, which are relative, log2-scaled abundance measures rather than absolute clinical concentrations (15). NPX values are derived by dividing sample counts by extension-control counts, followed by log2 transformation and adjustment for median extension-control counts, batch-specific median NPX, and assay-specific median NPX. All samples were processed within the UK Biobank Pharma Proteomics Project (UKB-PPP), which employed centralized quality control (QC) criteria including intra-plate coefficient of variation < 20% and sample-wise call rate > 70%. Proteins with > 50% missingness across the cohort were excluded from downstream analyses. For the remaining proteins, missing values primarily reflected measurements below the limit of detection (LOD) or technical failures; these were handled using Multiple Imputation by Chained Equations (MICE) with 10 imputed datasets and 20 iterations per dataset. The nine final model proteins (ANG, IL1R1, CXCL14, ESAM, PTGDS, HAVCR1, FGFR2, IGSF8, CCL3) exhibited call rates > 90%, well above the LOD threshold.

### Non-fatal MACE outcomes

The primary outcome was the first occurrence of non-fatal MACE, defined as non-fatal myocardial infarction or non-fatal stroke. All diagnoses were ascertained using ICD-10 codes and Read codes as detailed in **Supplementary Table 1** (16). Events were identified through linked hospital episode statistics, primary care records, and death registries. A recorded myocardial infarction or stroke was considered fatal if death occurred within 30 days of the event; such events were excluded from the non-fatal endpoint. The date of onset was defined as the earliest date at which any MACE component was recorded. Participants were followed from baseline until the first of non-fatal MACE diagnosis, death, or end of follow-up. Those with a history of non-fatal MACE at baseline and those lost to follow-up were excluded.

### Clinical variables

We extracted a comprehensive set of baseline characteristics from the UK Biobank to examine their relationship with incident non-fatal MACE in patients with diabetic kidney disease. Core demographics included age (years), sex (female/male), self-reported ethnicity (white vs. other ethnic groups) and the Townsend deprivation index. Socio-economic position was further characterized by an education score derived from qualifications attained. Anthropometric measurements comprised body-mass index (BMI, kg/m²), waist circumference (cm) and hip circumference (cm). Glycaemic and renal parameters comprised glycated hemoglobin (HbA1c, mmol/mol), plasma glucose (mmol/L), estimated glomerular filtration rate (eGFR, mL/min/1.73 m²), urinary albumin-to-creatinine ratio (ACR, mg/g) and serum urea (mmol/L). Lipid-related biomarkers included HDL-cholesterol, LDL-cholesterol, triglycerides, apolipoprotein B and lipoprotein A (mmol/L). Additional metabolic indices were serum albumin (g/L), alanine aminotransferase (U/L), calcium (mmol/L) and C-reactive protein (mg/L). Blood pressure values (systolic and diastolic, mmHg) were averaged from two seated readings. Lifestyle factors recorded current, never or previous smoking status and alcohol consumption. Polygenic risk scores for type 1 and type 2 diabetes were computed from imputed genotypes. Full variable definitions are listed in **Supplementary Table 2**.

### Cox regression analysis

As an exploratory discovery step, Cox proportional-hazards models with follow-up as the time scale were fitted to estimate hazard ratios (HRs) and 95% confidence intervals (CIs) for the association between each plasma protein and incident non-fatal MACE. This step was not part of the predictive model training. Because the initial screen involved 1,463 simultaneous protein-outcome tests and the training subset (70%, n ≈ 222) contained only approximately 48 events, restricting this screen to the training fold alone would have yielded inadequate statistical power and highly unstable protein lists under Benjamini–Hochberg correction. We therefore elected to use the full cohort for this hypothesis-generating phase, while reserving all predictive modeling decisions for the subsequent 70/30 split. Three sequential models were constructed: Model 1 (crude): no covariate adjustment. Model 2 (socio-demographic): adjusted for age at recruitment, sex, education score and Townsend deprivation index at recruitment. Model 3 (socio-demographic + metabolic): further adjusted for glycated hemoglobin (HbA1c), glucose, triglycerides, urea, BMI, eGFR, albumin-to-creatinine ratio, diastolic and systolic blood pressure (automated reading), waist circumference. The multicollinearity among candidate predictors was assessed using variance inflation factor(VIF). Benjamini–Hochberg correction was applied across all protein-outcome tests to control the false-discovery rate. Statistical significance was set at two-sided adjusted p < 0.05. These Cox models served as a hypothesis-generating screen to narrow the candidate pool from 1,463 proteins to a manageable set for downstream machine-learning validation. In *post-hoc* sensitivity analyses, we extended Model 3 by adding (1) diabetes duration, (2) diabetes duration+comorbidities (Hypertension, Dyslipidemia, Coronary heart disease, Heart failure), (3) diabetes duration+comorbidities+medications (Antihypertensive medication, Lipid-lowering medication and Insulin). Each of the core proteins was standardized per 1-SD and modeled separately. Missing clinical covariates were handled using multiple imputation by chained equations with 10 imputed datasets and 20 iterations; Cox estimates were pooled using Rubin’s rules.

### Functional enrichment

Proteins associated with non-fatal MACE risk (adjusted p < 0.05) across the three models were pooled, converted to Entrez gene symbols, and interrogated with the R packages clusterProfiler and org.Hs.eg.db for Gene Ontology (GO) annotation. Enrichment analyses covering biological process (BP), cellular component (CC) and molecular function (MF) were visualized with enrichplot. Statistical significance was declared at P < 0.05 and false-discovery rate (FDR) < 0.2; the top ten GO terms are displayed together with their intrinsic inter-relationships. KEGG pathway enrichment was performed with the enrichKEGG function using pvalueCutoff = 0.05 and qvalueCutoff = 0.2, and the resulting top fifteen pathways are presented with pairwise intersection information (17).

### Machine learning

Following the full-cohort Cox screening, the cohort was randomly partitioned into training (70%) and testing (30%) sets to prevent information leakage during model development. The 561 candidate proteins identified in the discovery phase (The union of the three models) constituted the input pool. All subsequent machine-learning feature selection, hyperparameter optimization, and model development, specifically the four-step pipeline (LASSO-Cox, random survival forest, Boruta, XGBoost-Cox) were performed strictly within the training set. The testing set remained entirely unseen until final performance assessment.

We constructed a systematic four-stage analytical architecture within the training data only. First, LASSO-Cox regularization was applied to the Cox-filtered protein panel; by shrinking most coefficients to exact zero it delivers a parsimonious set of high-impact predictors while handling high collinearity. The penalty parameter λ was chosen by ten-fold cross-validation at the 1-standard-error (λ_1_SE) criterion to ensure maximal sparsity (18). Second, a random survival forest with 1000 trees, maximum depth of 8 nodes and minimum terminal size of 5 events was trained on the retained markers, its ensemble of decorrelated trees captures non-linear hazard functions and provides permutation-based importance rankings, allowing further culling of noise variables (19). Third, the Boruta algorithm was executed with 100 maximum iterations, p-value threshold 0.01 and Bonferroni-type multiple-comparison adjustment; only proteins that consistently outperform their permuted shadow counterparts are deemed confirmed relevant and carried forward, thereby guarding against false positives (20). Finally, we employed an XGBoost-Cox model with a learning rate of 0.01, a maximum tree depth of 3, a subsample rate of 0.8, and 500 boosting rounds to achieve optimal feature sparsification. By integrating gradient boosting with Newton-type optimization, this model flexibly accounts for complex feature interactions and intricate time-to-event patterns. Furthermore, the subsequent SHAP decomposition mathematically quantifies the marginal contribution of each protein to every individual’s predicted risk trajectory (21). Features exhibiting stable mean absolute SHAP values across ten-fold cross-validation were considered robust and retained for clinical interpretation.

### Survival analyses and simulated trajectories of key protein

The machine-learning-derived proteins were dichotomized at the optimal Youden-index threshold, and Kaplan–Meier curves were constructed to compare non-fatal MACE incidence between high- and low-expression groups. To mitigate the impact of death as a competing risk, we employed the Fine and Gray sub-distribution hazard model, which allows for the estimation of the cumulative incidence of non-fatal MACE while accounting for the competing risk of death. Longitudinal trajectories of plasma proteins were modeled over a 10-year period prior to the index date using a landmark analysis framework matched by follow-up time. Smoothing curves were used to visualize temporal trends; Mann-Kendall tests assessed monotonicity. Mixed-effects models were applied to test time-group interactions.

### Establishment of predictive model and webpage deployment

Receiver operating characteristic analyses evaluated predictive accuracy of the protein signature alone and in combination with demographic indicators (age, sex, ethnicity, education, Townsend index) and clinical traits (HbA1c, glucose, lipids, BMI, blood pressure, renal markers). Five sequential models were compared: protein-only, protein plus demographics, protein plus clinical factors, protein plus demographics and clinical factors, demographics plus clinical factors. DeLong tests and bootstrap analyses with 2,000 iterations assessed AUC differences (22). To mitigate overfitting, the cohort was randomly partitioned into training (70%) and testing (30%) sets, with repeated cross-validation performed. Systematic algorithm benchmarking screened 101 machine learning combinations using the *Mime* framework (R package) to identify the optimal modeling approach. The evaluation metrics prioritized Harrell’s C-index and time-dependent AUC at 10 years to account for follow-up duration and censoring. The CoxBoost + Elastic Net framework, demonstrating the most consistent C-index performance and stable time-dependent AUCs across both training and independent testing cohorts, was selected as the final model. This approach ensures the clinical interpretability of the protein signature within a longitudinal time-to-event framework.

## Results

### Participants’ characteristics

The flowchart of participant selection was shown in **Supplementary Figure 1**. Among 317 DKD adults free of non-fatal MACE at baseline, 68 (21.5%) developed a non-fatal event during follow-up (38 myocardial infarction, 34 stroke). **Table 1** compares baseline characteristics between participants who remained event-free during follow-up (non-MACE group, N = 249) and those who experienced a non-fatal MACE (MACE group, N = 68). Age, sex, ethnicity and socio-economic markers were well balanced: mean age ≈ 62 years, 44% female, >90% white, similar Townsend deprivation index and education score (all P ≥ 0.20). Anthropometry and standard metabolic indices also overlapped—BMI ≈ 33 kg/m², waist 107–108 cm, HDL-cholesterol ≈ 1.15 mmol/L, triglycerides ≈ 2.5 mmol/L, blood pressure ≈ 145/79 mmHg, indicating comparable adiposity and cardiovascular risk factor burden (all P ≥ 0.29). Glycaemic control was modestly worse in non-fatal MACE cases (HbA1c 60.1 vs 55.0 mmol/mol, P = 0.078), accompanied by lower albumin (42.9 vs 43.8 g/L, P = 0.072) and higher LDL-cholesterol (2.82 vs 2.62 mmol/L, P = 0.133), although these differences did not reach conventional significance. Renal function parameters (eGFR, ACR, urea) and inflammatory marker C-reactive protein were almost identical (P ≥ 0.29). In addition, the baseline prevalence of coronary heart disease in the non-fatal MACE group was higher than that in the non-MACE group [21/68 (30.9%) vs. 43/249 (17.3%), P = 0.017], and the duration of diabetes was also longer [12.5 (11.5) years vs. 17.2 (13.6) years, P = 0.005]. No significant differences were observed in the prevalence of hypertension, dyslipidemia, heart failure, or baseline use of antihypertensive, lipid-lowering, or insulin therapy between groups. Taken together, apart from subtle deterioration in glucose handling and marginally lower serum albumin, individuals who subsequently experienced non-fatal MACE were clinically indistinguishable at baseline, underscoring the need for more sensitive predictive biomarkers.

**Table 1:** Baseline characteristics of UK Biobank participants included in the study.

Participants’ characteristics	Participants who remained event-free during follow-up (N=249)	Participants who experienced non-fatal MACE(N=68)	P.overall	Participants who experienced Myocardial infarction (N=38)	P.overall	Participants who experienced Stroke(N=34)	P.overall
Age (years)	62.9 (5.79)	62.0 (6.75)	0.324	61.5 (7.36)	0.296	63.0 (5.73)	0.761
Gender			0.264		0.845		0.166
Female	109 (43.8%)	24 (35.3%)		17 (44.7%)		10 (29.4%)	
Male	140 (56.2%)	44 (64.7%)		21 (55.3%)		24 (70.6%)	
Ethnicity (white)	232 (93.2%)	64 (94.1%)		35 (92.4%)		32 (93.8%)	
Townsend deprivation index	-0.59 (3.36)	0.01 (3.51)	0.206	-0.70 (3.39)	0.645	0.61 (3.56)	0.069
Education score	21.0 (19.0)	21.5 (19.0)	0.862	18.0 (14.5)	0.225	24.0 (23.1)	0.499
BMI (kg/m²)	32.9 (5.90)	32.8 (5.79)	0.829	33.4 (5.93)	0.599	32.6 (5.74)	0.800
Enhanced PRS for T1D	0.13 (1.18)	0.26 (1.20)	0.606	0.70 (1.17)	0.054	-0.11 (1.17)	0.330
Enhanced PRS for T2D	0.64 (0.86)	0.54 (0.99)	0.624	0.80 (0.95)	0.365	0.24 (0.95)	0.116
HbA1c (mmol/mol)	55.0 (22.0)	60.1 (19.7)	0.078	60.7 (18.7)	0.129	58.9 (20.1)	0.421
eGFR (mL/min/1.73 m²)	54.4 (18.0)	52.6 (24.1)	0.571	53.2 (28.9)	0.843	52.4 (17.7)	0.590
ACR (mg/g)	39.3 (71.2)	35.9 (52.3)	0.700	33.5 (36.0)	0.484	36.9 (64.7)	0.890
Urea (mmol/L)	9.24 (3.92)	9.87 (4.36)	0.293	10.2 (4.71)	0.290	9.52 (3.80)	0.804
Glucose (mmol/L)	7.82 (3.93)	8.52 (4.76)	0.300	8.65 (4.38)	0.331	8.30 (5.12)	0.703
HDL (mmol/L)	1.14 (0.31)	1.16 (0.37)	0.701	1.17 (0.42)	0.754	1.14 (0.27)	0.911
Triglycerides (mmol/L)	2.42 (1.37)	2.57 (1.35)	0.436	2.43 (1.07)	0.912	2.71 (1.53)	0.317
Apolipoprotein B (mmol/L)	0.83 (0.23)	0.88 (0.24)	0.118	0.90 (0.25)	0.106	0.87 (0.23)	0.449
Lipoprotein A (mmol/L)	45.8 (48.0)	46.0 (48.0)	0.977	41.3 (44.9)	0.622	51.6 (49.7)	0.556
LDL (mmol/L)	2.62 (0.86)	2.82 (0.93)	0.133	2.77 (0.75)	0.380	2.89 (1.07)	0.184
Albumin (g/L)	43.8 (3.42)	42.9 (3.18)	0.072	42.3 (3.13)	0.012	43.6 (3.03)	0.995
Alanine aminotransferase (U/L)	25.5 (12.8)	24.6 (14.4)	0.641	21.9 (11.5)	0.077	27.9 (17.0)	0.352
Calcium (mmol/L)	2.38 (0.11)	2.38 (0.12)	0.722	2.36 (0.12)	0.327	2.39 (0.12)	0.600
Alcohol status:			0.467		0.502		0.744
Current	195 (78.3%)	56 (82.4%)		31 (81.6%)		29 (85.3%)	
Never	23 (9.24%)	8 (11.8%)		5 (13.2%)		3 (8.82%)	
Previous	30 (12.0%)	4 (5.88%)		2 (5.26%)		2 (5.88%)	
Smoking status:			0.467		0.867		0.211
Current	19 (7.63%)	4 (5.88%)		3 (7.89%)		1 (2.94%)	
Never	117 (47.0%)	28 (41.2%)		16 (42.1%)		14 (41.2%)	
Previous	112 (45.0%)	35 (51.5%)		19 (50.0%)		18 (52.9%)	
Diastolic blood pressure (mmHg)	79.0 (10.3)	78.2 (12.5)	0.628	76.8 (11.8)	0.281	80.3 (13.0)	0.501
Systolic blood pressure (mmHg)	145 (19.0)	147 (21.9)	0.537	150 (19.5)	0.210	146 (23.7)	0.991
Waist circumference (cm)	107 (14.2)	108 (14.3)	0.654	108 (14.3)	0.546	108 (14.5)	0.629
Hip circumference (cm)	111 (12.2)	111 (12.4)	0.615	111 (12.3)	0.941	111 (12.8)	0.737
C reactive protein (mg/L)	5.07 (8.21)	4.95 (5.77)	0.893	4.69 (4.82)	0.679	5.37 (6.54)	0.772
Diabetes duration, years	12.5 (11.5)	17.2 (13.6)	0.005	20.6 (14.6)	<0.001	13.6 (10.9)	0.420
Hypertension	198 (79.5%)	55 (80.9%)	0.866	28 (73.7%)	0.401	31 (91.2%)	0.160
Dyslipidemia/high cholesterol	99 (39.8%)	24 (35.3%)	0.575	14 (36.8%)	0.859	10 (29.4%)	0.266
Coronary heart disease	43 (17.3%)	21 (30.9%)	0.017	13 (34.2%)	0.026	9 (26.5%)	0.236
Heart failure	12 (4.8%)	6 (8.8%)	0.236	2 (5.3%)	1.000	4 (11.8%)	0.111
Antihypertensive medication	204 (81.9%)	57 (83.8%)	0.858	30 (78.9%)	0.656	29 (85.3%)	0.811
Lipid-lowering medication	192 (77.1%)	50 (73.5%)	0.524	27 (71.1%)	0.417	24 (70.6%)	0.396
Insulin	72 (28.9%)	20 (29.4%)	1.000	11 (28.9%)	1.000	10 (29.4%)	1.000

Data are presented as mean (standard deviation) for continuous variables and n (%) for categorical variables. P values for continuous variables were derived from independent samples t-tests (comparisons: Non-fatal MACE group vs. event-free group; Myocardial infarction group vs. event-free group; Stroke group vs. event-free group). P values for categorical variables (Gender, Alcohol status, Smoking status) were derived from chi-square tests. All P values are two-sided. The “P.overall” columns for each outcome group (Non-fatal MACE, Myocardial infarction, Stroke) represent the P value for the comparison between that specific case group and the event-free group. For example, the P values in the “Myocardial infarction” column compare the Myocardial infarction group to the event-free group.

### Identifying proteins associated with non-fatal MACE

**Figure 1:**
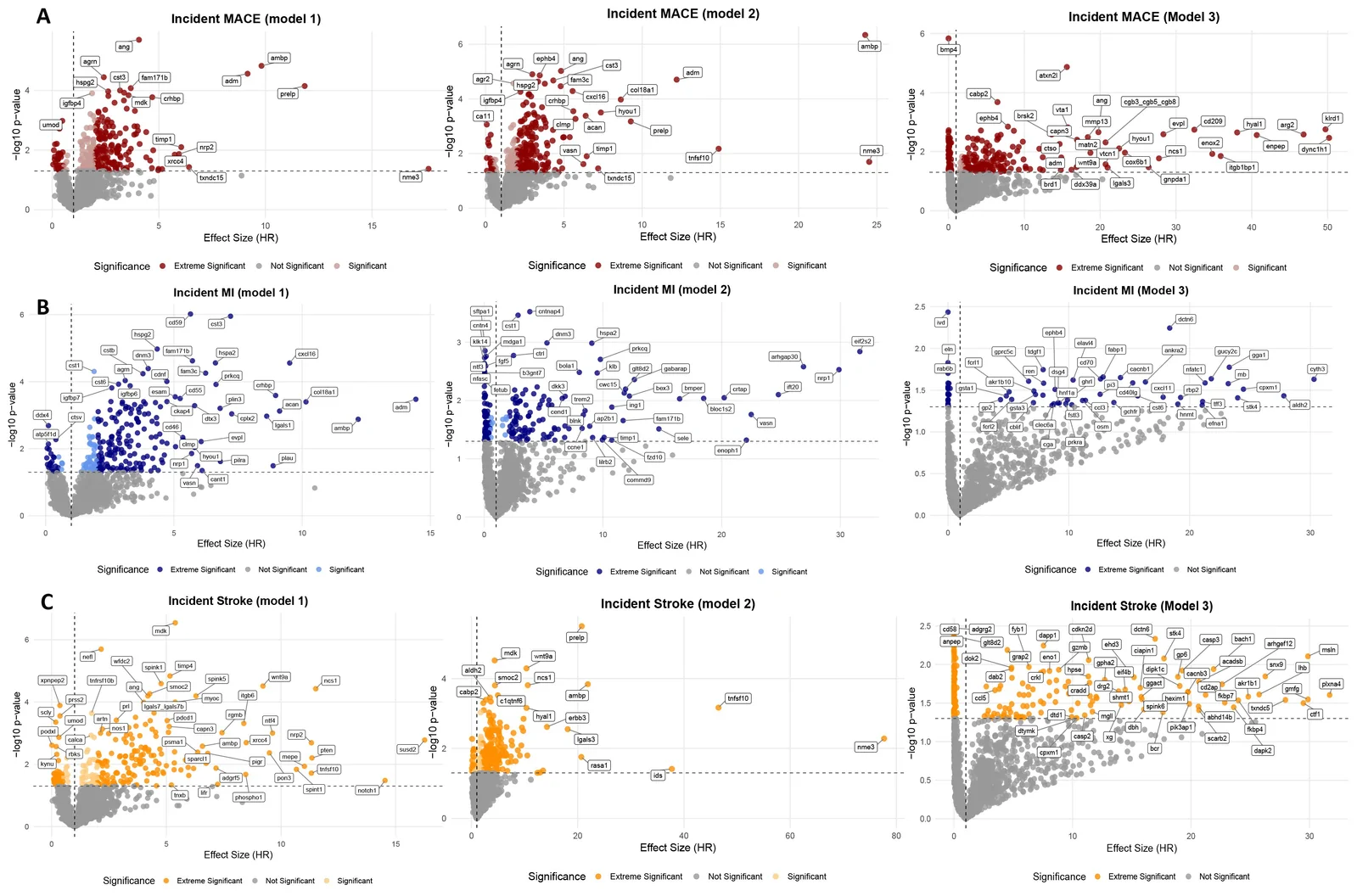
Association of plasma proteins with incident MACE. The median follow-up time for the study population was 10.2 years (interquartile range: 8.2-12.1 years. **(A)** Volcano plot depicting the association of 1,463 plasma proteins with incident MACE. Model 1 (crude): no covariate adjustment. Model 2 (socio-demographic): adjusted for age at recruitment, sex, education score and Townsend deprivation index at recruitment. Model 3 (socio-demographic + metabolic): further adjusted for glycated hemoglobin (HbA1c), glucose, triglycerides, urea, BMI, eGFR, albumin-to-creatinine ratio, diastolic and systolic blood pressure (automated reading), waist circumference. **(B)** Volcano plot depicting the association of 1,463 plasma proteins with incident MI and **(C)** with incident stroke. MACE, Major Adverse Cardiovascular Events. MI, myocardial infarction.(HR > 2.0 or HR < 0.5) & adjusted P < 0.05 for “extreme significant” and (HR > 1.2 or HR < 0.8) & adjusted P < 0.05 for “significant”. Benjamini–Hochberg correction was applied across all protein-outcome tests to control the false-discovery rate.

In the full-cohort exploratory analysis, among 1,463 plasma proteins examined, 241 proteins showed the strongest crude associations with non-fatal MACE, with effect sizes ranging from HR 1.26 to 7.65 (all adjusted P < 0.05). After adjustment for socio-demographic factors (age at recruitment, sex, education score, Townsend deprivation index), 343 proteins showed a significant association with non-fatal MACE. Further correction for metabolic variables (HbA1c, glucose, lipid profile, BMI, blood pressure, eGFR, ACR, urea) yielded 135 proteins and even larger effect estimates (HR 2.93–9.92). A summary of the three models identified a total of 561 significant proteins, with 14 proteins found at the intersection, which are ANG, FGFR2, PTGDS, EPHB4, REG1A, FABP6, CCL27, GPHA2, IGFBP6, PI3, IGFBP3, COL6A3, CXCL14, and CST6. Volcano plots (**Figure 1A**) visualized these associations using thresholds of (HR > 2.0 or HR < 0.5) & adjusted P < 0.05 for “extreme significant” and (HR > 1.2 or HR < 0.8) & adjusted P < 0.05 for “significant”. Analyses restricted to myocardial infarction (**Figure 1B**) identified the same metabolic-adjusted top 10 proteins, while stroke-specific associations (**Figure 1C**) were dominated by RABGAP1L, IGDCC4, DTD1, and others. The intersection proteins of the three models of non-fatal MACE and the MI/Stroke models further intersect at three proteins: ANG, FGFR2, PTGDS with average adjusted HR above 2.0.

### Functional enrichment

The enriched pathway based on 561 significant proteins (a summary of the three cox models) was shown in **Supplementary Figure 2**. GO terms related to biological processes (BP), such as “positive regulation of MAPK cascade” and “leukocyte migration”, were significant. These terms are involved in signaling pathways that modulate inflammation and immune responses—processes known to play pivotal roles in the pathogenesis of DKD and its progression to non-fatal MACE. The “positive regulation of cell adhesion” and “leukocyte migration” were connected, indicating a coordinated role in immune cell trafficking and tissue repair. KEGG pathway enrichment analysis provided additional insights into the inflammation and immune responses. The specific ranking of KEGG was shown in **Supplementary Table 3**.

### Machine learning

**Figure 2:**
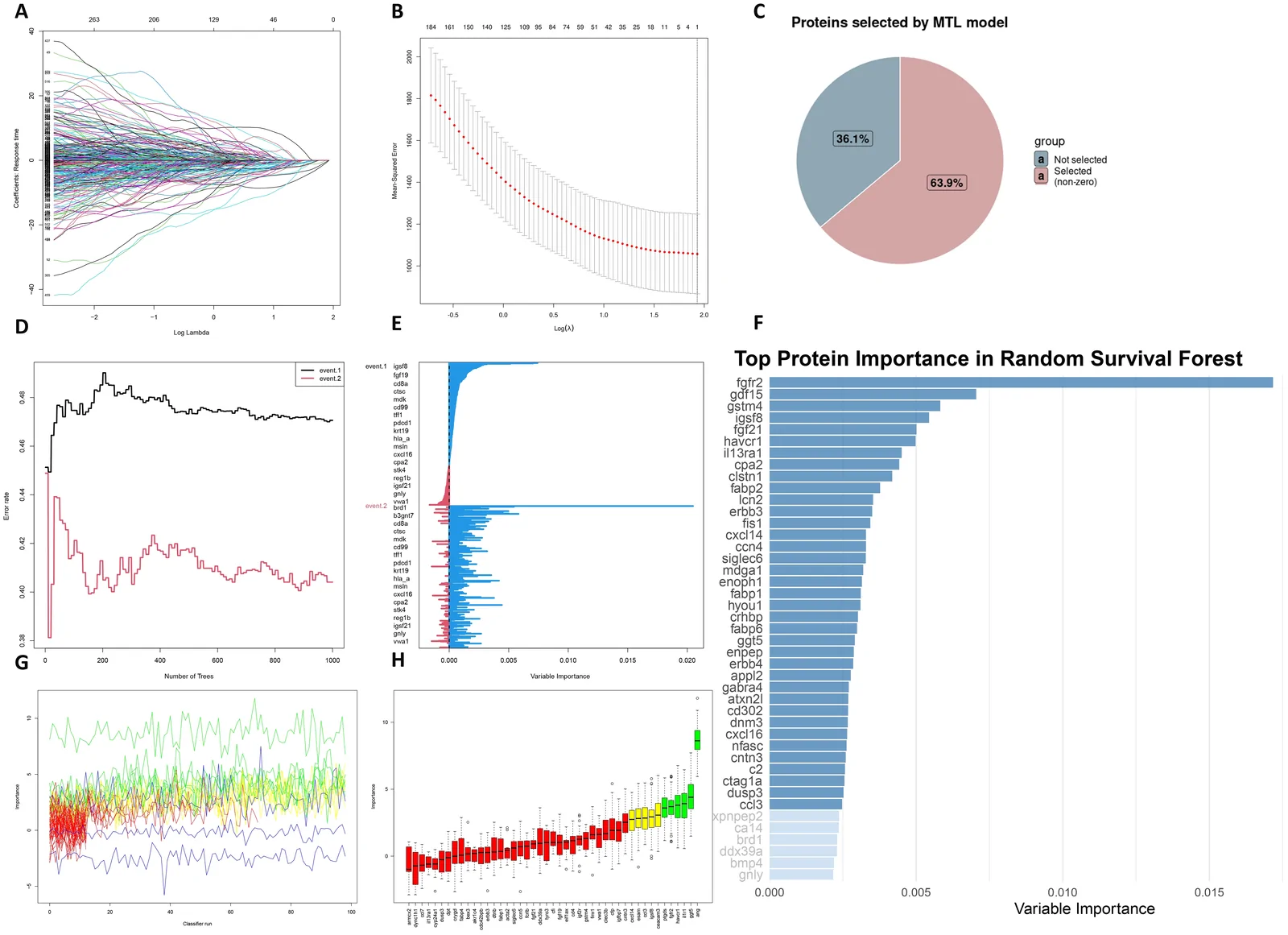
Key protein predictor selection for MACE outcome in the training set (70% of total samples, 40 events in 222 patients). **(A)** Coefficient paths of the top-ranked proteins across log(λ) values in the LASSO-Cox model. **(B)** Ten-fold cross-validation error curve; vertical dashed lines indicate λ at minimum error (λ.min) and the 1-standard-error rule (λ.1SE). **(C)** Multi-task learning confirming 63.9% of the LASSO-selected proteins as significant. **(D–F)** Random Survival Forest (RSF) outputs highlighting 37 key proteins, including error rate by number of trees **(D)**, variable importance rankings **(E)**, and minimal depth distributions **(F)**. **(G, H)** Boruta algorithm results ultimately selecting 11 core features after comprehensive variable importance evaluation, showing importance scores of confirmed, rejected, and shadow features **(G)** and final feature selection status **(H)**.

The 561 candidate proteins identified from the full-cohort Cox screening were subsequently input into the four-step machine-learning pipeline, which was executed strictly within the training set. LASSO-Cox (**Figures 2A, B**) identified optimal protein predictors through iterative coefficient minimization, with multi-task learning (**Figure 2C**) confirming 63.9% of the LASSO-selected proteins as significant. Random Survival Forest (**Figures 2D–F**) highlighted 37 key proteins. Boruta algorithm (**Figures 2G, H**) ultimately selected 11 core features after comprehensive variable importance evaluation.

**Figure 3:**
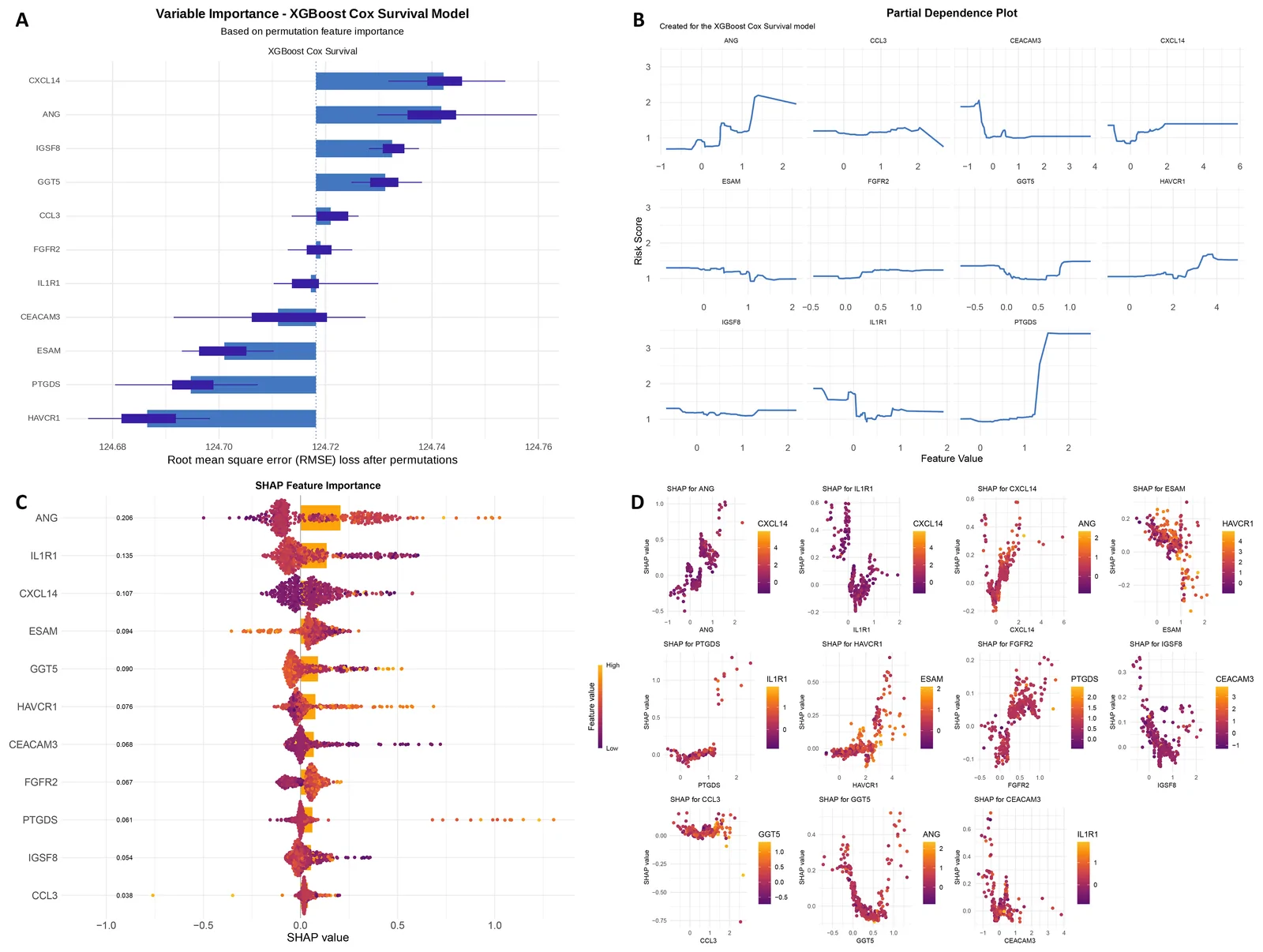
XGBoost-Cox model interpretation and SHAP-based evaluation of the 11 Boruta-selected proteins. **(A)** XGBoost gain-based contribution scores ranking the 11 proteins (CXCL14, ANG, IGSF8, GGT5, CCL3, FGFR2, IL1R1, CEACAM3, ESAM, PTGDS, and HAVCR1). Longer bars indicate greater contribution to the average predicted risk score. **(B)** Partial Dependence Plots (PDP) showing the marginal effect of ANG, CXCL14, and PTGDS on predicted risk. Risk scores for ANG and CXCL14 increased non-linearly as their NPX values rose, whereas PTGDS exhibited a sharp threshold effect once its value exceeded approximately 1.5. **(C)** Protein importance ranking according to mean absolute SHAP values (ANG, IL1R1, CXCL14, ESAM, GGT5, HAVCR1, CEACAM3, FGFR2, PTGDS, IGSF8, and CCL3). Each point represents an individual observation, with color indicating protein concentration (red, high; blue, low); positive SHAP values denote increased predicted risk. **(D)** SHAP dependence plot for ANG, colored by CXCL14 expression level. ANG SHAP values increased non-linearly with its concentration, with the highest risk contributions observed when CXCL14 was concurrently elevated, indicating a synergistic interaction between these two proteins in amplifying MACE risk.

XGBoost-Cox and SHAP values were used to rank the 11 Boruta-selected proteins. These 11 proteins were ranked according to their contribution scores as CXCL14, ANG, IGSF8, GGT5, CCL3, FGFR2, IL1R1, CEACAM3, ESAM, PTGDS and HAVCR1. About half of the proteins could increase the average predicted risk score (**Figure 3A**). Their bar charts of CXCL14 and ANG extended the farthest to the right, indicating their importance for maintaining the accuracy of the model’s predictions. The Partial Dependence Plots indicated that the risk scores of ANG and CXCL14 increased as the characteristic values rise, while for PTGDS, the risk score showed a sharp threshold effect after the characteristic value reaches approximately 1.5. (**Figure 3B**). The importance ranking according to SHAP was ANG, IL1R1, CXCL14, ESAM, GGT5, HAVCR1, CEACAM3, FGFR2, PTGDS, IGSF8, and CCL3 (**Figure 3C**). Each protein showed a positive contribution to the individual prediction results (SHAP values were greater than 0). When the concentration of ANG increased, its SHAP value showed a non-linear increase; and it occurred simultaneously with the high expression of CXCL14. These two proteins mutually amplified each other’s risk of disease (**Figure 3D**). The distribution maps of the SHAP markers for typical non-fatal MACE patients and those without MACE were shown in **Supplementary Figure 3**.

### Survival analyses and simulated trajectories of key protein

**Figure 4:**
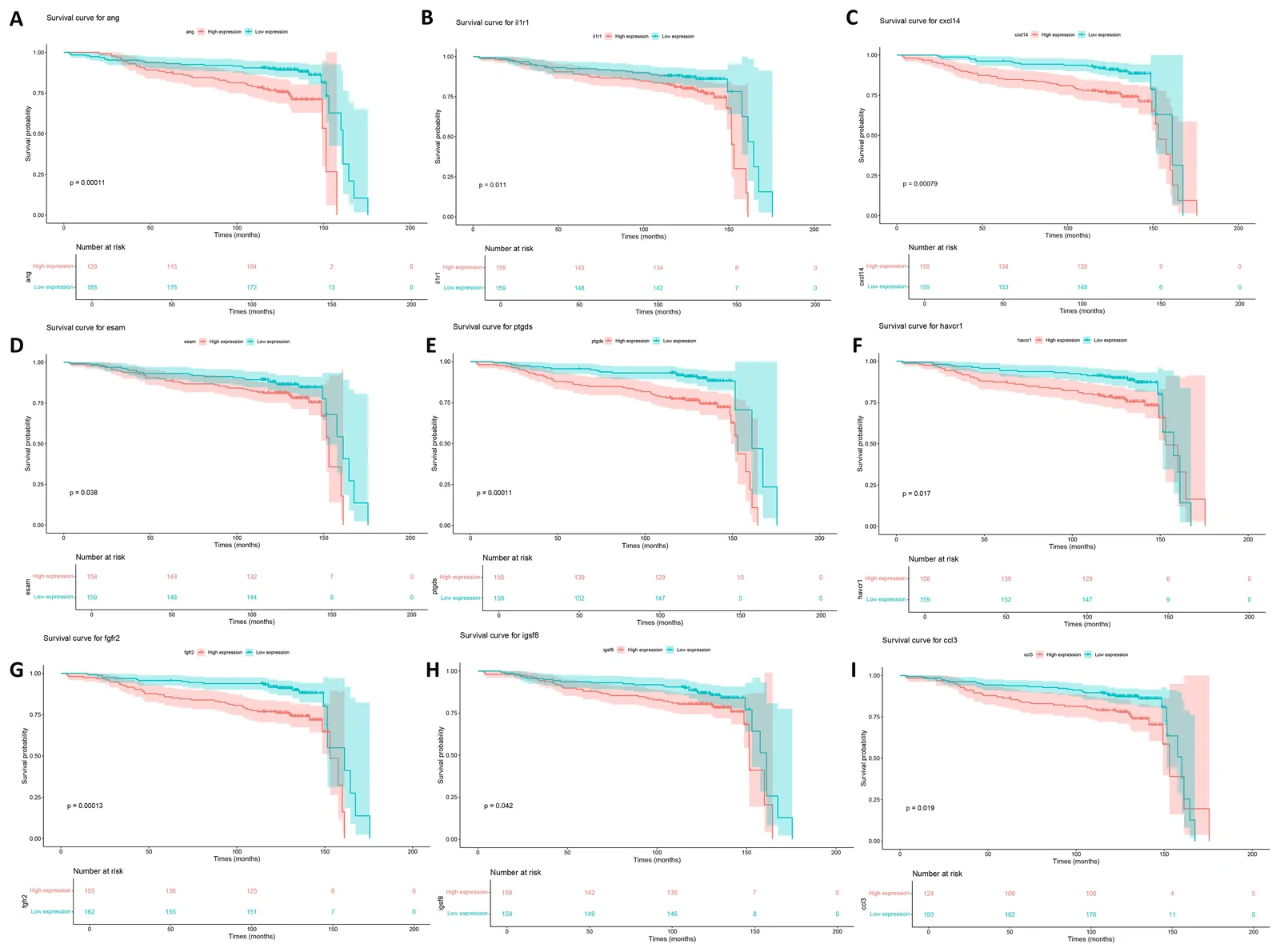
Survival analysis of key proteins for MACE risk stratification in 317 DKD patients. Researchers categorized baseline levels of these proteins into high and low expression groups based on optimal threshold values derived from the Youden index and conducted survival analysis. Kaplan-Meier curves revealed significant differences in MACE-free survival rates between high and low expression groups for nine key proteins **(A)** ANG (threshold=0.423, HR = 3.88[2.33 - 6.48], P < 0.001, strongest association), **(B)** IL1R1 (threshold=0.289, HR = 2.42[1.25 - 4.69]), **(C)** CXCL14 (threshold=0.326[1.03 - 1.70], HR = 1.32), **(D)** ESAM (threshold=0.567, HR = 2.19[1.31 - 3.65]), **(E)** PTGDS (threshold=0.663, HR = 2.65[1.39 - 5.06]), **(F)** HAVCR1 (threshold=1.514, HR = 1.38[1.12 - 1.69]), **(G)** FGFR2 (threshold=0.396, HR = 1.71[1.02 - 2.44]), **(H)** IGSF8 (threshold=0.474, HR = 1.26[1.21 - 2.05]), and **(I)** CCL3 (threshold=0.693, HR = 1.37[1.01 - 2.90]).

To assess the relevance of key proteins in the progression from DKD to non-fatal MACE, we categorized baseline levels of these proteins into high and low expression groups based on optimal threshold values derived from the Youden index and conducted survival analysis. Kaplan-Meier curves revealed significant differences in MACE-free survival rates between high and low expression groups for nine key proteins: ANG (threshold = 0.423), IL1R1 (threshold = 0.289), CXCL14 (threshold = 0.326), ESAM (threshold = 0.567), PTGDS (threshold = 0.663), HAVCR1 (threshold = 1.514), FGFR2 (threshold = 0.396), IGSF8 (threshold = 0.474), and CCL3 (threshold = 0.693). Individuals with higher baseline levels of ANG exhibited a significantly elevated risk of non-fatal MACE, with a hazard ratio (HR) of 3.88 (95% confidence interval: 2.33 - 6.48; p < 0.001) (**Figure 4A**). Similarly, elevated levels of IL1R1 (**Figure 4B**), CXCL14 (**Figure 4C**), ESAM (**Figure 4D**), PTGDS (**Figure 4E**), HAVCR1 (**Figure 4F**), FGFR2 (**Figure 4G**), IGSF8 (**Figure 4H**), and CCL3 (**Figure 4I**) were associated with increased MACE risk, with HRs of IL1R1: 2.42 (95% CI: 1.25 - 4.69), CXCL14: 1.32 (95% CI: 1.03 - 1.70), ESAM: 2.19 (95% CI: 1.31 - 3.65), PTGDS: 2.65 (95% CI: 1.39 - 5.06), HAVCR1: 1.38 (95% CI: 1.12 - 1.69), FGFR2: 1.71 (95% CI: 1.01 - 2.90), IGSF8: 1.26 (95% CI: 1.21 - 2.05) and CCL3: 1.37 (95% CI: 1.01 - 2.90), respectively. However, GGT5 (threshold = 0.289) and CEACAM3 (threshold = 0.079) showed no significant association with non-fatal MACE risk (**Supplementary Figure 4**). They were therefore excluded, leaving the final set of 9 proteins.

**Figure 5:**
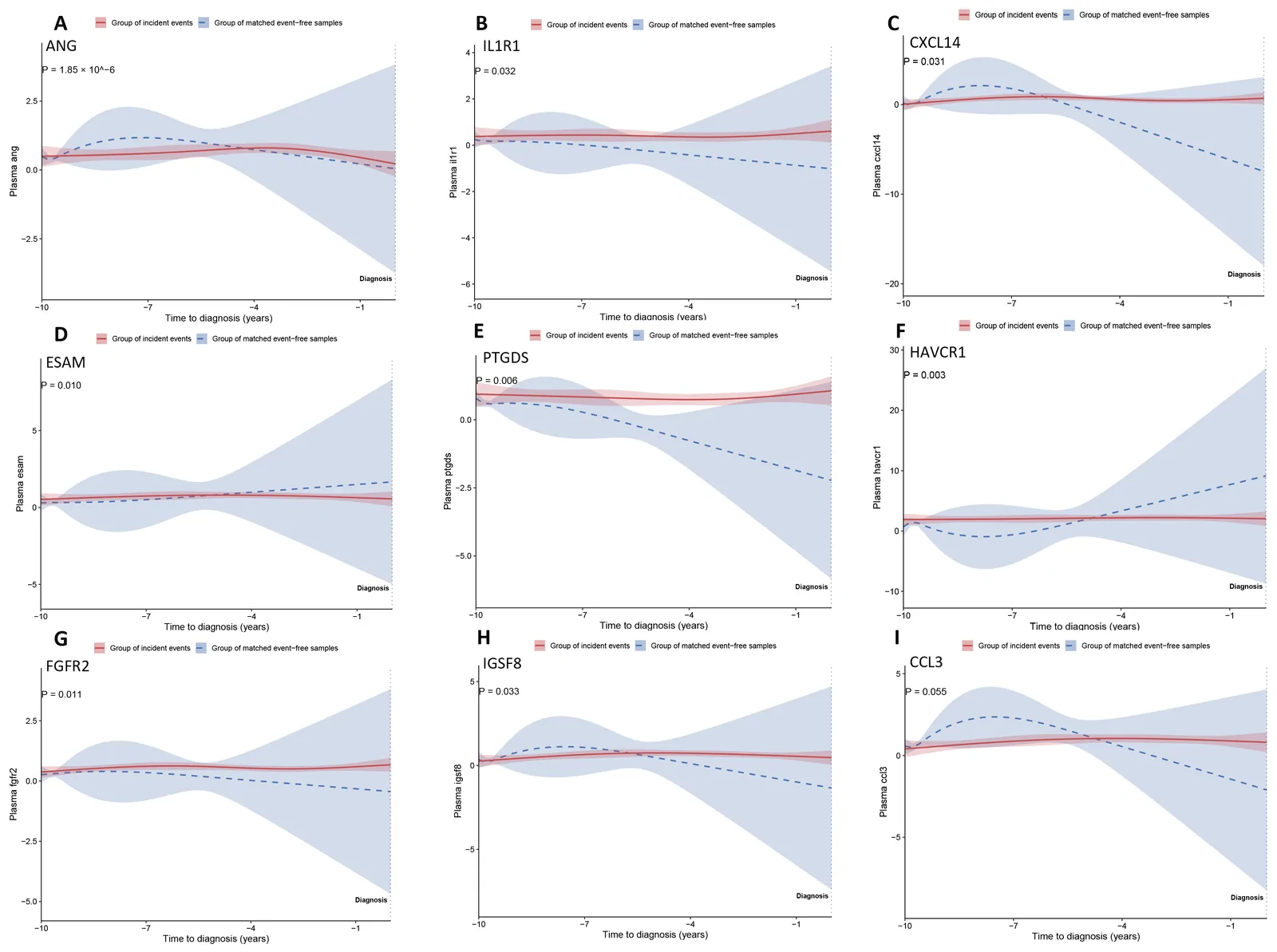
Simulated trajectories of plasma proteins preceding MACE diagnosis in DKD patients. Dynamic changes of plasma **(A)** ANG, **(B)** IL1R1, **(C)** CXCL14, **(D)** ESAM, **(E)** PTGDS, **(F)** HAVCR1, **(G)** FGFR2, **(H)** IGSF8 and **(I)** CCL3 before MACE. Trajectories analysis matched 68 MACE event samples to 249 event-free samples by age (± 2 years), sex, hypertension and hyperlipidemia. Red curves: MACE event samples; blue curves: MACE event-free samples. Annual mean protein concentrations with 95% CIs. Mixed-effects models tested trajectory differences (two-sided P values, no multiple testing correction).

Simulated trajectories revealed significant between-group differences for all nine proteins (P < 0.05) (**Figure 5**). IL1R1 and CXCL14 remained consistently elevated throughout the 10-year period. In contrast, IGSF8, FGFR2, CCL3, and HAVCR1 showed no initial differences but gradually became significantly elevated as the event approached. Sensitivity analyses revealed that CCL3 was the only biomarker whose 95% confidence interval crossed the unity line following the inclusion of diabetes duration (Model 3 + duration). For the remaining eight proteins, the estimated hazard ratios (HRs) and their corresponding 95% confidence intervals remained above the null line (HR = 1.00), irrespective of the adjustment strategy, spanning from the fully adjusted baseline model (Model 3) to sequential sensitivity models including disease duration, comorbidities, and medications. Among these, ANG (Angiogenin) exhibited the most pronounced risk effect(**Supplementary Figure 5**).

### Predictive accuracy of plasma proteins and clinical indicators

**Figure 6:**
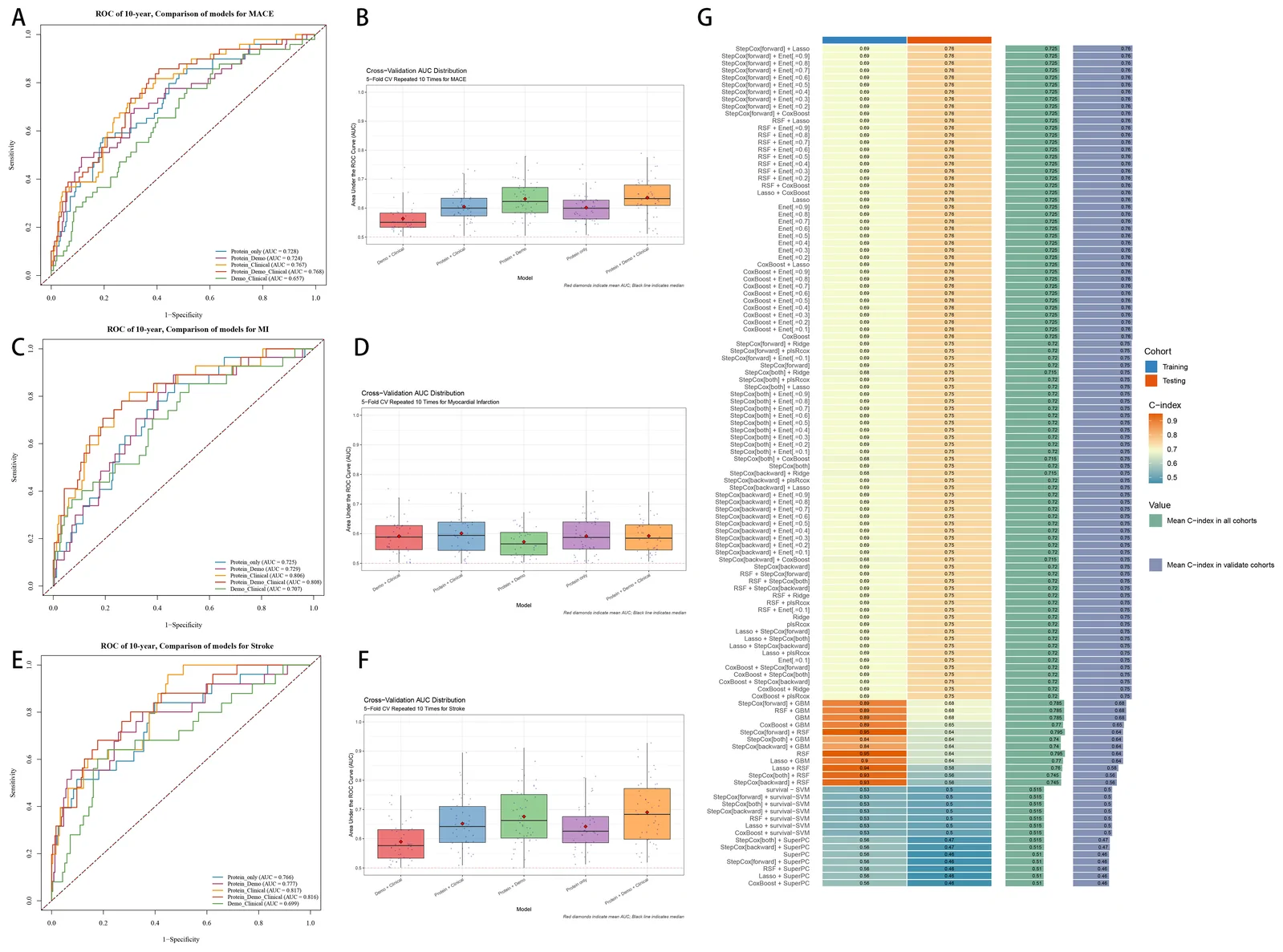
Performance evaluation and benchmarking of the validated protein signature and clinical factors. ROC curves and AUC distributions are presented for the MACE outcome **(A, B)**, MI outcome **(C, D)**, and stroke outcome **(E, F)**. Five models were compared: (1) Protein-only; (2) Protein + Demographics; (3) Protein + Clinical factors; (4) Protein + demographics + clinical factors and (5)Demographics + clinical factors. **(A, B)** The model integrating proteins, demographics, and clinical variables achieved the highest area under the curve (AUC = 0.768) for predicting MACE outcomes, in the repeated cross-validation analysis, the model also demonstrated higher and more stable AUC values, reinforcing its superior generalizability. **(C–F)** The model integrating proteins, demographics, and clinical variables achieved the highest AUC for predicting MI (0.808) and stroke outcome(0.816). The repeated cross-validation analysis showed the stability of the five models was approximately the same. **(G)** Systematic benchmarking of machine learning algorithms via *Mime* package. Among 101 algorithm combinations screened, the CoxBoost + Elastic framework was selected as the final optimal model due to its exceptional stability and high C-index performance across both training (C-index = 0.69) and testing (C-index = 0.76) set.

**Figure 7:**
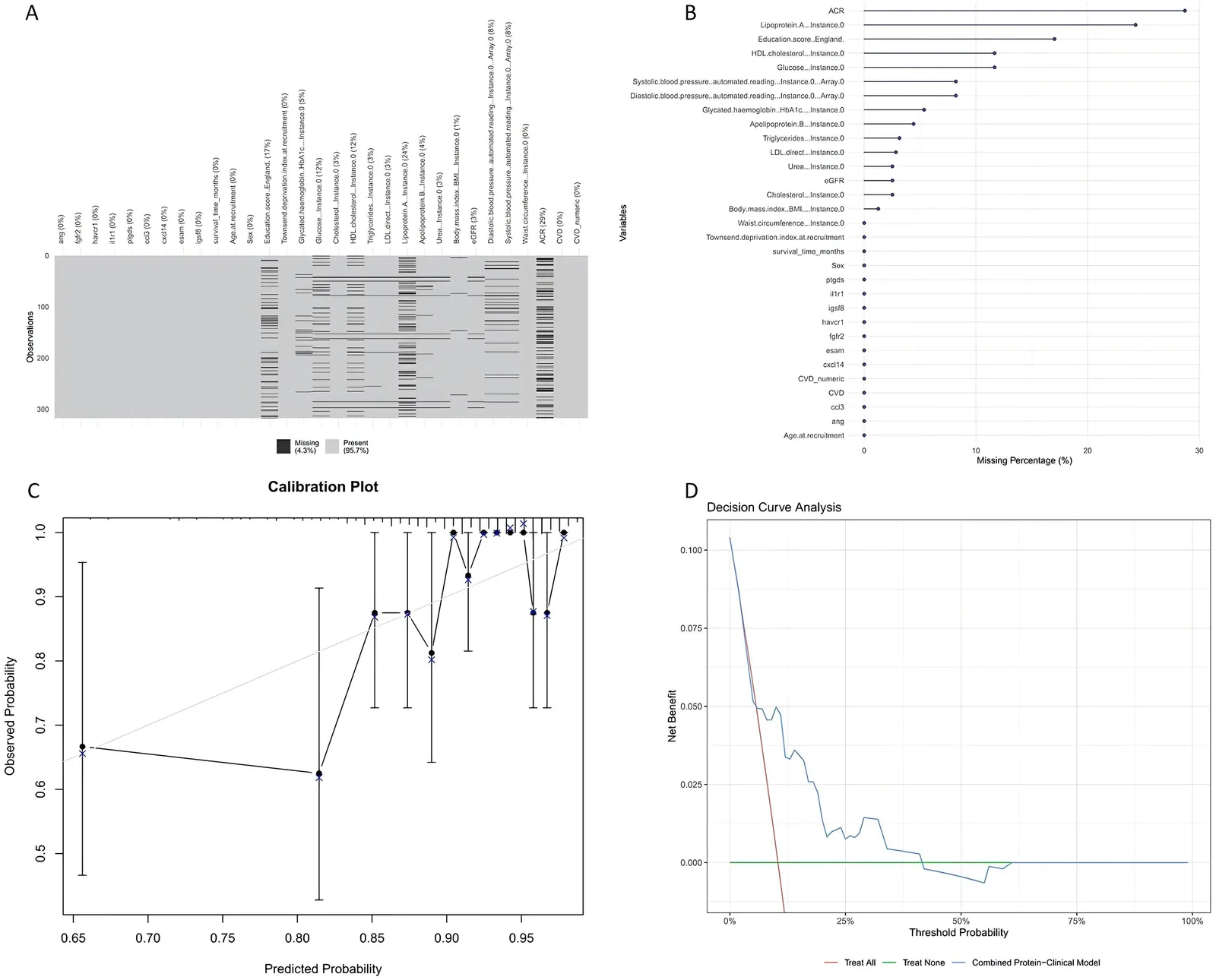
Visualization of pre-interpolation data and final model calibration. **(A, B)** The amount of missing data before interpolation was all below 30%. **(C)** The horizontal axis (Predicted Probability) represents the model-estimated risk of MACE; the vertical axis (Observed Probability) represents the actual risk estimated via Kaplan-Meier. In the high-risk region (>0.85), the curve closely aligns with the ideal line, with multiple points clustered above 0.9, indicating that the model is highly accurate in identifying extremely high-risk patients. **(D)** The red line (Treat All) represents the assumption that all DKD patients receive intensive intervention as if they were all at high risk. The green line (Treat None) represents the assumption that no additional intervention is given to any patient, resulting in a net benefit of zero. The blue line (Combined Model) represents decisions guided by the protein-clinical combined model. Within the range of approximately 5% to 45% on the horizontal axis (decision threshold probability), the blue line consistently lies above both the red and green lines. This indicates that if clinicians consider a 5% to 45% risk threshold as warranting intervention, using the model to guide decisions provides significant additional net benefit compared to either “treating all” or “treating none”.

The nine key proteins identified were selected as significant model variables and subjected to collinearity analysis alongside common clinical indicators. Comprehensive performance evaluation of the validated protein and clinical signatures were presented in **Figure 6**. ROC analyses compared five sequential modeling approaches across non-fatal MACE outcomes (**Figures 6A, B**), MI (**Figures 6C, D**), and stroke (**Figures 6E, F**). For non-fatal MACE (**Figure 6A**), the model integrating proteins, demographics, and clinical variables achieved the highest area under the curve (AUC = 0.768). The protein-only model yielded an AUC of 0.728, while adding demographic or clinical features progressively improved performance. The demographic-plus-clinical model (without proteins) had the lowest AUC (0.657), highlighting the added value of proteomic data. **Figure 6B** displayed the cross-validation AUC distribution, where the full model again exhibited the highest median AUC and narrowest interquartile range, indicating superior stability. Similar patterns were observed for MI (**Figures 6C, D**) and stroke (**Figures 6E, F**), where the combined model consistently outperformed the other approaches. Systematic algorithm benchmarking via 101 method combinations (**Figure 6G**) identified CoxBoost + Elastic Net Model as the optimal framework, achieving C-index = 0.69 in the training cohort and 0.76 in the testing cohort. This performance exceeded alternative approaches including naive Bayes, support vector machines, and k-nearest neighbors, validating the analytical pipeline selection. The missing values in the original data of the model source do not exceed 30% (**Figures 7A, B**). The calibration assessment and decision curve analysis were shown in **Figures 7C, D**. The model demonstrated favorable calibration in high-risk patients and yielded positive net clinical benefit across decision thresholds of 5% to 45%.

### Web application deployment

**Figure 8:**
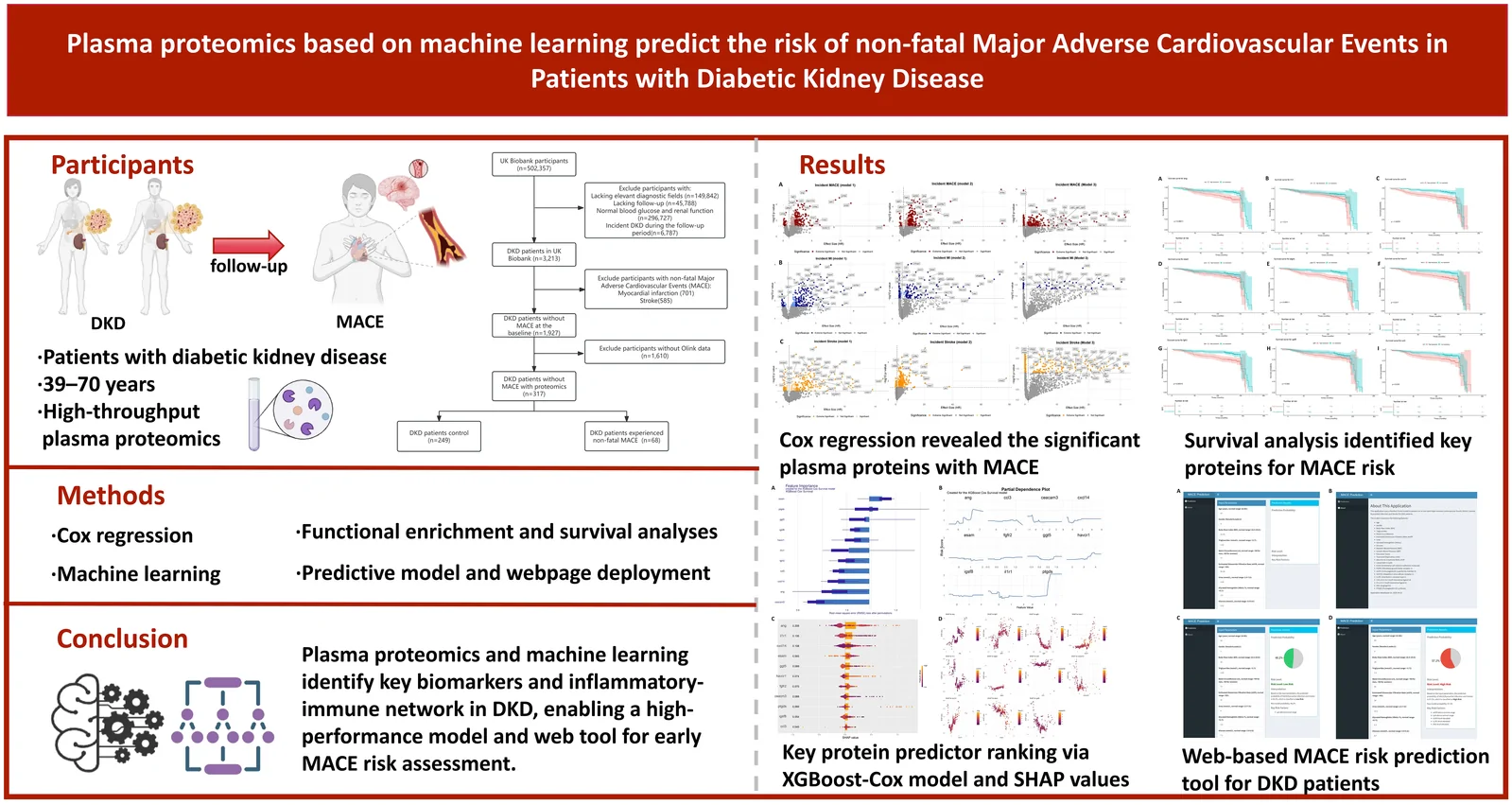
Graphic abstract.

The deployed web-based prediction tool was illustrated in **Supplementary Figure 6**. The interface enables clinicians to input NPX value for the 9 serum proteins and 19 clinical indicators, with real-time calculation of non-fatal MACE outcome risk probability. The application displays model parameters, protein reference ranges, and biomarker annotations to support clinical interpretation. This digital platform translates the research findings into an accessible, evidence-based decision support system for personalized DKD management. The complete process of the article was shown in **Figure 8**.

## Discussion

In this study, we systematically identified and validated nine plasma protein biomarkers (ANG, IL1R1, CXCL14, ESAM, PTGDS, HAVCR1, FGFR2, IGSF8, and CCL3) for incident non-fatal MACE in patients with DKD. These proteins demonstrated consistent predictive value across all analytical workflows, including Cox regression, multiple machine learning-based feature selection methods, and Kaplan-Meier survival analysis, effectively predicting non-fatal MACE risk during follow-up.

Previous studies have shown that plasma proteomics can improve cardiovascular risk prediction beyond traditional risk factors, although the composition of the resulting protein panels has varied substantially. In a UK Biobank population without previous cardiovascular disease, Royer et al. measured 2,919 proteins and derived a 114-protein data-driven model that provided only modest incremental improvement over traditional risk factors for non-fatal MACE prediction (23). Hoogeveen et al. similarly developed a 50-protein primary-prevention model in which GDF15 made the largest contribution, while natriuretic peptide-related markers, including NT-proBNP and BNP, were also prominent (24). Among patients with stable coronary heart disease, Ganz et al. developed a nine-protein score for myocardial infarction, stroke, heart failure, or death. This score improved discrimination over a refitted Framingham model, although its overall accuracy remained moderate (25). Collectively, these studies support the incremental predictive value of plasma proteomics while demonstrating that panel composition is strongly influenced by the study population, clinical endpoint, assay platform, and modeling strategy.

Diabetes-specific studies further illustrate this context dependence. In the KORA cohorts, Luo et al. measured 233 cardiovascular and inflammatory proteins. Among participants with baseline T2D, a four-protein-enriched model produced only a modest improvement over the Framingham variables (ΔC-index=0.017), and the principal incident CHD-associated proteins validated in the T2D stratum were CXCL9 and IL2RA (26). In a high-risk angiographic population with diabetes, Mohebi et al. evaluated a four-biomarker panel comprising KIM-1, NT-proBNP, osteopontin, and TIMP-1 (27). Notably, KIM-1 is encoded by HAVCR1 and therefore represents direct overlap with our final panel. By comparison, our panel comprising ANG, IL1R1, CXCL14, ESAM, PTGDS, HAVCR1, FGFR2, IGSF8, and CCL3 may preferentially capture cardiorenal injury, endothelial adhesion, chemokine signaling, and inflammatory remodeling in DKD. The limited marker-level overlap with previous panels should therefore not be interpreted as contradictory evidence, but may instead reflect differences in the target population and predicted outcome.

The behavior of GDF15 and NT-proBNP in our analyses further illustrates how study design can shape the selected signature. GDF15 was prominent in the random survival forest importance analysis but was not retained after the subsequent Boruta and XGBoost-Cox stages, which required stable support across complementary selection procedures. Its exclusion therefore indicates insufficient selection stability in this dataset rather than an absence of cardiovascular relevance. This instability may have been amplified by the limited number of events, particularly the 40 events in the training set. In addition, chronic renal and metabolic stress in patients with DKD may reduce the incremental discrimination provided by a broadly responsive stress marker such as GDF15. NT-proBNP was not among the 561 proteins entering the machine-learning pipeline. The present study analyzed 1,463 study-available proteins, whereas Royer et al. evaluated 2,919 proteins (23). Its absence from the final panel should therefore not be interpreted as evidence that NT-proBNP lacks prognostic value in DKD. Moreover, our endpoint was restricted to non-fatal myocardial infarction and non-fatal stroke and excluded cardiovascular death, outcome for which GDF15 and natriuretic peptides often have particularly strong prognostic value. Thus, the DKD-specific sampling frame, non-fatal MACE definition, Olink analyte availability, limited event count, and sequential feature-selection pipeline may all have contributed to the divergence from previous panels. Future external validation should include prespecified head-to-head measurements of GDF15 and NT-proBNP to determine whether the nine-protein panel provides predictive information beyond these established comparators.

The three significantly enriched biological processes in the GO analysis, positive regulation of MAPK cascade, leukocyte migration, and positive regulation of cell adhesion, collectively formed the basis for the establishment of a chronic inflammatory micro-environment in the cardiovascular system of DKD patients. In DKD patients, long-term hyperglycemia, accumulation of uremic toxins and intolerance to hypoxic environments continuously stimulated the activation of the MAPK pathway in renal and vascular endothelial cells (28). Activated MAPK track with the expression and secretion of pro-inflammatory factors such as tumor necrosis factor-α (TNF-α) and interleukin-6 (IL-6) by phosphorylating transcription factors including AP-1 and NF-κB (29). The leukocytes crossed the endothelial barrier, entered the subendothelial layer, differentiated into macrophages, and phagocytosed oxidized low-density lipoprotein (ox-LDL), thereby participating in the formation of the lipid core in atherosclerosis (30).

Individual proteins within this study model demonstrated unique diagnostic potential. ANG (angiogenin), FGFR2 (fibroblast growth factor receptor 2), and PTGDS (prostaglandin D2 synthase) were not only proteins with high diagnostic value for non-fatal MACE but also key markers for the common risk of concurrent myocardial infarction (MI) and stroke. The three were linked to the development of cardiovascular complications in DKD patients and mirrored the underlying pathological processes of vascular injury, inflammation activation, and tissue repair imbalance.

ANG refers to angiogenin (ribonuclease 5, encoded by ANG), not angiopoietin. It showed the strongest association with non-fatal MACE (HR, 3.88; 95% CI, 2.33-6.48) and ranked highest in the SHAP analysis. Angiogenin is a stress-responsive ribonuclease involved in endothelial signaling, angiogenesis, and vascular remodeling. Experimental evidence suggests that it activates endothelial nitric oxide synthase through phosphoinositide 3-kinase/Akt signalling (31). Circulating angiogenin has been associated with glycaemic control (32) and DKD progression (33). In type 2 diabetes, higher angiogenin remained associated with incident MACE after adjustment for kidney function and albuminuria (34). It has also been linked to adverse outcomes after acute coronary syndrome (35). Urinary angiogenin increases during renal epithelial stress and correlates with kidney impairment and proteinuria (36). Thus, circulating ANG may reflect combined metabolic, renal, and vascular stress in DKD. However, this association requires external validation and does not establish a causal role in non-fatal MACE. Other literature indicates that FGFR2 is associated with the modulation of vascular smooth muscle cell (VSMC) phenotypes. In the context of DKD, uremic toxins have been reported to stimulate FGFR2, which tracks with a synthetic phenotype via PI3K-Akt-mTOR signaling (37). These altered VSMCs are characterized by enhanced proliferation, migration, and secretion of MMP-9, a process documented to correlate with atherosclerotic plaque instability through collagen degradation and subsequent myocardial infarction risk (38). Concurrently, the literature suggests that cerebral vessel remodeling and basement membrane degradation linked to MMP-9 are tied to elevated risks of both ischemic and hemorrhagic stroke (39). Within our study, the predictive value of FGFR2 potentially reflects its clinical alignment with plaque instability and vascular remodeling, processes that underlie both myocardial infarction and stroke.

Regarding PTGDS, the key enzyme for prostaglandin D2 synthesis, previous functional studies show it is involved in NLRP3 inflammasome activation and IL-1β/IL-18 release upon glycosylation and macrophage uptake (40). It also displays interactions with HDL implicated in the facilitation of reverse cholesterol transport. Consequently, the inclusion of PTGDS in our model likely captures the interplay between inflammation and dyslipidemia, two hallmarks of DKD-related cardiovascular risk. In DKD, glycosylated PTGDS has been observed to correlate with altered HDL structure and function, which corresponds to reduced cholesterol clearance and antioxidative capacity (41). These changes closely parallel the progression of atherosclerosis in coronary arteries and the worsening of cerebral endothelial injury and inflammatory responses, thereby reflecting increased clinical susceptibilities to both MI and stroke (42).

Currently, MACE risk assessment for patients with DKD has primarily relied on traditional cardiovascular risk factors, such as the cardio-metabolic variables (43), SYNTAX score (based on the anatomical characteristics of coronary arteries) (44) and triglyceride-glucose index (45). However, these models have all lacked large-scale proteomic selection and did not have sufficiently long follow-up periods. Li et al. developed a predictive model for incident CHD in T2D patients by integrating nine causal proteins (including PCSK9, NRP1, and CD27) and achieved an AUC of 0.816 (46). Additionally, Kachhawa et al. demonstrated that dyslipidemia combined with elevated cystatin C levels could serve as predictive markers for chronic kidney disease in T2DM patients, highlighting the potential of integrating multiple biomarker classes for improved risk stratification (47). Furthermore, uremic toxins including p-cresyl sulfate and indoxyl sulfate have been identified as promising biomarkers for predicting renal function across GFR categories (48). In our analysis, the combined model incorporating nine plasma proteins (ANG, FGFR2, PTGDS, etc.) together with demographic and clinical variables achieved a time-dependent AUC of 0.768 for MACE prediction. Notably, our model also attained a C-index of 0.768 for the composite MACE outcome, which is comparable to the CHD-specific model, despite MACE representing a broader and more heterogeneous endpoint (myocardial infarction and stroke) than CHD alone. Our model constructed in this study integrated DKD-specific proteins, including ANG and FGFR2, along with renal function indicators such as eGFR and ACR, demonstrating higher predictive accuracy in the DKD population. This tool was especially suitable for primary healthcare institutions, where clinicians, without the need for complex machine learning expertise, could input 24 basic patient indicators to rapidly obtain risk assessment results, facilitating the clinical cycle of early screening and precise intervention.

Limitations and future directions of this study included several aspects: First, the sample lacked racial representativeness. The UK Biobank cohort was predominantly composed of white individuals (over 90%), and the applicability of the model in DKD patients of other ethnicities such as Asian and African needed further validation. Second, the relatively small sample size of 317 DKD patients and the modest number of MACE events (n = 68) may impact the stability of the machine-learning model and the generalizability of the findings. Small sample sizes can increase the risk of overfitting and reduce the precision of performance estimates. While regularization, repeated cross-validation, and strict feature-selection pipelines were employed to mitigate this risk, the event-per-variable ratio remains suboptimal. Consequently, the predictive metrics reported should be interpreted as promising internal validation estimates rather than definitive population parameters. Also, due to the limited number of MACE outcome events, we did not conduct further validation of the model (NRI/IDI, calibration slope and intercept). We adopted a two-stage analysis process to identify the positive protein. The workflow, while carefully structured to minimize information leakage, does not fully eliminate the possibility that the exploratory Cox screen on the full cohort introduced some degree of optimism into the candidate protein pool. Another notable limitation of this study is the absence of external validation. While the UK Biobank offers an unparalleled resource for proteomic discovery, other large-scale cohorts such as CHARLS and NHANES currently lack comparable plasma proteomics data, making independent external validation difficult at present. Accordingly, our findings should be regarded as promising, internally validated discovery results that would benefit from confirmation in independent cohorts before broader clinical adoption. We hope that as more proteomic datasets become publicly available, future studies will be able to conduct such external validation.

Our model was trained on NPX values. The log2-scaled abundance was generated by the Olink PEA platform, rather than on absolute clinical concentrations. This distinction is not merely semantic: NPX reflects the composite signal of antibody-recognizable epitopes, which may pool full-length proteins, cleaved fragments, and isoforms indiscriminately. For ANG and FGFR2, both of which exhibit splice variants with divergent biological activity, the assay cannot resolve which molecular species drive the observed association with MACE. Nor can it exclude cross-reactivity with homologous proteins or matrix interference from hemolyzed or lipemic samples, despite the UKB-PPP pre-analytical QC. These constraints imply that the web tool, while immediately deployable for research and exploratory clinical use, should be interpreted as a platform-specific decision aid rather than a universal calculator. Its predictions are validated only for Olink Explore 3072 outputs processed through the UKB-PPP normalization pipeline; direct portability to SomaScan, mass spectrometry, or even differently normalized Olink batches would require bridging studies with certified reference materials. We view this not as a fatal flaw but as a defining feature of contemporary proteomic medicine: the path from discovery assay to clinical chemistry is necessarily iterative, and our model represents a calibrated waypoint rather than a final destination.

## Conclusion

We integrated plasma proteomics with clinical and demographic factors to successfully construct a robust predictive model for non-fatal MACE in DKD patients. Core biomarkers including ANG, FGFR2 and PTGDS were identified, shedding light on the molecular mechanisms of DKD-related cardiovascular complications. The CoxBoost + Elastic Net framework exhibited an acceptable performance and was deployed as an interactive web tool for clinical accessibility.

## Data Availability

The original contributions presented in the study are included in the article/**Supplementary Material**. Further inquiries can be directed to the corresponding authors.
