# Supplementary material for: Discovery and validation of a multi-protein panel for predicting non-fatal major adverse cardiovascular events in diabetic kidney disease
Source: Front Endocrinol (Lausanne). 2026 Aug 27;17:1883523. doi: 10.3389/fendo.2026.1883523 (PMC13558116; doi:10.3389/fendo.2026.1883523)
Supplement: Supplementary file 1 [file SupplementaryFile1.docx]

**Supplementary Table 1** Disease codes in UK Biobank

|  | ICD-10 | ICD-9 | OPCS-4 | self-reported | READ2 for primary care data |
| --- | --- | --- | --- | --- | --- |
| Diabetic Kidney Disease | E10.2, E11.2, E14.2, N08.3, N18 | 2503, 585 | M01, X40 | 20002(1192,1193,1194,1519,1607),  20004(1195,1580,1581,1582) | C109G, C1116, K791. |
| Stroke | I60, I61, I63, I64 | 430,431,434,436 | L35、L71 | 1081, 1086, 1583 | G6..., G61.., G64.. |
| Myocardial Infarction | I21-I23, I241, I252 | 410-412 | K49、K50、K75 | 1075 | G30.., G311., G35.. |
| Diabetes | E08, E09, E10, E11, E12, E13 | 250 | C82.1 |  | C10.. (T1), C11.. (T2) |
| The table lists the specific diagnostic codes from various classification systems used to identify cases of Diabetic Kidney Disease, Stroke, Myocardial Infarction, and Diabetes in the UK Biobank dataset. **ICD-10**: International Classification of Diseases, Tenth Revision; **ICD-9**: International Classification of Diseases, Ninth Revision; **OPCS-4**: Office of Population Censuses and Surveys Classification of Interventions and Procedures, Fourth Revision. | | | | | |

**Supplementary Table 2** Extraction of clinical variables from the UK Biobank

| Category | Field ID | Description |
| --- | --- | --- |
| Baseline characteristics | 21022 | Age at recruitment |
|  | 33 | Date of birth |
|  | 31 | Sex |
|  | 21000 | Ethnic background |
|  | 54 | UK Biobank assessment centre |
|  | 53 | Date of attending assessment centre |
|  | 21003 | Age when attended assessment centre |
| Body composition | 21001 | Body mass index |
|  | 23128 | Trunk fat mass |
|  | 48 | Waist circumference |
|  | 49 | Hip circumference |
|  | 23105 | Basal metabolic rate |
|  | 50 | Standing height |
|  | 21002 | Weight |
| Disease definition | 42007 | Source of stroke report |
|  | 42006 | Date of stroke |
|  | 42001 | Source of myocardial infarction report |
|  | 42000 | Date of myocardial infarction |
|  | 41270 | Diagnoses - ICD10 |
|  | 41280 | Date of first in-patient diagnosis - ICD10 |
|  | 41202 | Diagnoses - main ICD10 |
|  | 41203 | Diagnoses - main ICD9 |
|  | 41204 | Diagnoses - secondary ICD10 |
|  | 41205 | Diagnoses - secondary ICD9 |
| Smoking and drinking | 20116 | Smoking status |
|  | 20161 | Pack years of smoking |
|  | 1239 | Current tobacco smoking |
|  | 1249 | Past tobacco smoking |
|  | 2644 | Light smokers, at least 100 smokes in lifetime |
|  | 20117 | Alcohol drinker status |
|  | 20403 | Amount of alcohol drunk on a typical drinking day |
|  | 1558 | Alcohol intake frequency |
|  | 100580 | Alcohol consumed |
| Blood pressure | 93 | Systolic blood pressure, manual reading |
|  | 94 | Diastolic blood pressure, manual reading |
|  | 4080 | Systolic blood pressure, automated reading |
|  | 4079 | Diastolic blood pressure, automated reading |
| Lost to follow-up and death | 191 | Date lost to follow-up |
|  | 190 | Reason lost to follow-up |
|  | 40000 | Date of death |
|  | 40000 | Date of death |
|  | 40001 | Underlying (primary) cause of death: ICD10 |
|  | 40002 | Contributory (secondary) causes of death: ICD10 |
| Comorbidities | 131286 | Date I10 first reported (essential (primary) hypertension) |
|  | 131294 | Date I15 first reported (secondary hypertension) |
|  | 130706 | Date E10 first reported (insulin-dependent diabetes mellitus) |
|  | 130708 | Date E11 first reported (non-insulin-dependent diabetes mellitus) |
|  | 6152 | Blood clot, DVT, bronchitis, emphysema, asthma, rhinitis, eczema, allergy diagnosed by doctor |
|  | 6150 | Vascular/heart problems diagnosed by doctor |
|  | 2443 | Diabetes diagnosed by doctor |
|  | 20544 | Mental health problems ever diagnosed by a professional |
|  | 3627 | Age angina diagnosed |
|  | 4056 | Age stroke diagnosed |
|  | 3992 | Age emphysema/chronic bronchitis diagnosed |
|  | 29976 | Age diabetes diagnosed |
| Medications | 6177 | Medication for cholesterol, blood pressure or diabetes |
|  | 6153 | Medication for cholesterol, blood pressure, diabetes, or take exogenous hormones |
|  | 20003 | Treatment/medication code |
| Blood biochemistry | 30620 | Alanine aminotransferase |
|  | 30600 | Albumin |
|  | 30640 | Apolipoprotein B |
|  | 30710 | C-reactive protein |
|  | 30680 | Calcium |
|  | 30690 | Cholesterol |
|  | 30700 | Creatinine |
|  | 30720 | Cystatin C |
|  | 30740 | Glucose |
|  | 30750 | Glycated haemoglobin (HbA1c) |
|  | 30760 | HDL cholesterol |
|  | 30780 | LDL direct |
|  | 30790 | Lipoprotein A |
|  | 30870 | Triglycerides |
|  | 30670 | Urea |
|  | 30890 | Vitamin D |
| Proteomics | [30903](https://biobank.ndph.ox.ac.uk/showcase/field.cgi?id=30903) | [UKB-PPP Consortium selected participant](https://biobank.ndph.ox.ac.uk/showcase/field.cgi?id=30903) |
|  | [30900](https://biobank.ndph.ox.ac.uk/showcase/field.cgi?id=30900) | [Number of proteins measured](https://biobank.ndph.ox.ac.uk/showcase/field.cgi?id=30900) |
|  | [30901](https://biobank.ndph.ox.ac.uk/showcase/field.cgi?id=30901) | [Plate used for sample run](https://biobank.ndph.ox.ac.uk/showcase/field.cgi?id=30901) |
|  | [30902](https://biobank.ndph.ox.ac.uk/showcase/field.cgi?id=30902) | [Well used for sample run](https://biobank.ndph.ox.ac.uk/showcase/field.cgi?id=30902) |
|  | Protein name | olink_instance_0 |
|  | Protein name | olink_instance_2 |
|  | Protein name | olink_instance_3 |
| The table lists the specific UK Biobank data-field IDs (first column) and their corresponding descriptions (second column) for the biochemical biomarkers and proteomics data used in this study. Data-fields under “Proteomics” include sample processing information and the measured protein levels, where “Protein name” is a placeholder for the actual names of the ~3,000 proteins assayed in the UK Biobank Pharma Proteomics Project (UKB-PPP), and “olink_instance_x” denotes the specific measurement occasion (visit or assay plate). ICD: International classification of diseases; HDL: High-density lipoprotein; LDL: Low-Density Lipoprotein; | | |

**Supplementary Table 3** KEGG pathway

|  | category | subcategory | Description | GeneRatio | P  value | p.adjust | qvalue |
| --- | --- | --- | --- | --- | --- | --- | --- |
| hsa04060 | Environmental Information Processing | Signaling molecules and interaction | Cytokine-cytokine receptor interaction | 45/337 | 7.58E-17 | 2.16E-14 | 1.93E-14 |
| hsa04061 | Environmental Information Processing | Signaling molecules and interaction | Viral protein interaction with cytokine and cytokine receptor | 19/337 | 1.79E-09 | 2.56E-07 | 2.29E-07 |
| hsa04640 | Organismal Systems | Immune system | Hematopoietic cell lineage | 18/337 | 1.19E-08 | 1.13E-06 | 1.01E-06 |
| hsa04514 | Environmental Information Processing | Signaling molecules and interaction | Cell adhesion molecules | 20/337 | 9.63E-07 | 6.86E-05 | 6.13E-05 |
| hsa04151 | Environmental Information Processing | Signal transduction | PI3K-Akt signaling pathway | 30/337 | 1.40E-05 | 7.98E-04 | 7.14E-04 |
| hsa04010 | Environmental Information Processing | Signal transduction | MAPK signaling pathway | 26/337 | 2.48E-05 | 1.18E-03 | 1.05E-03 |
| hsa04610 | Organismal Systems | Immune system | Complement and coagulation cascades | 11/337 | 2.77E-04 | 1.09E-02 | 9.76E-03 |
| hsa04512 | Environmental Information Processing | Signaling molecules and interaction | ECM-receptor interaction | 11/337 | 3.06E-04 | 1.09E-02 | 9.76E-03 |
| hsa03265 | Genetic Information Processing | Information processing in viruses | Virion - Ebolavirus | 12/337 | 9.22E-04 | 6.28E-04 | 1.99E-02 |
| hsa04940 | Human Diseases | Endocrine and metabolic disease | Type I diabetes mellitus | 7/337 | 8.48E-04 | 2.22E-02 | 1.98E-02 |
| hsa04014 | Environmental Information Processing | Signal transduction | Ras signaling pathway | 19/337 | 8.56E-04 | 2.22E-02 | 1.98E-02 |
| hsa04210 | Cellular Processes | Cell growth and death | Apoptosis | 13/337 | 1.19E-03 | 2.83E-02 | 2.53E-02 |
| The table lists Kyoto Encyclopedia of Genes and Genomes (KEGG) pathways significantly enriched among the set of differentially expressed or associated proteins/genes identified in the study. For each pathway, the table provides its KEGG identifier (hsa), category, subcategory, and Description. The enrichment analysis was performed using the clusterProfiler and enrichKEGG R package. GeneRatio represents the ratio of the number of input genes mapped to a given pathway to the total number of input genes that could be mapped to any KEGG pathway. The P value was calculated using a hypergeometric test. The p.adjust and qvalue columns represent the P value adjusted for multiple testing using the Benjamini-Hochberg (FDR) method. Pathways are sorted by the P value in ascending order (most to least significant). | | | | | | | |

**Supplementary Figure 1** Flowchart of participant selection

**Supplementary Figure 2** Enriched Gene Ontology (GO) terms and KEGG pathways in DKD-to-MACE progression. (A-C) GO-BP analyses highlight inflammation- and immune-related processes (e.g., MAPK cascade regulation, leukocyte migration, cell adhesion) and their coordinated roles in immune cell dynamics and cellular function modulation. (D-F) KEGG pathway analyses identify core signaling pathways (e.g., Cytokine-cytokine receptor interaction, PI3K-Akt) critical for DKD vascular complications, and visualize their complex crosstalk with cellular adhesion/immune responses, as well as key molecules mediating pathway interactions.

**Supplementary Figure 3** The distribution maps of the SHAP markers for typical MACE patients and those without MACE. (A-C)Three randomly selected DKD patients who did not experience MACE. (D-F)Three randomly selected DKD patients who experienced MACE.

**Supplementary Figure 4** Survival analysis of GGT5 and CEACAM3 for MACE risk stratification in DKD. (A )GGT5 (threshold = 0.289) and (B) CEACAM3 (threshold = 0.079) showed no significant association with MACE risk.

Supplementary Figure 5. Sensitivity analyses for the nine-protein panel.Forest plot illustrating the hazard ratios (HRs) and 95% confidence intervals (CIs) per 1-standard deviation (SD) higher Olink NPX values for the prediction of major adverse cardiovascular events (MACE) across different adjustment models (N = 317, number of MACE events = 68) on a logarithmic scale. The plus sign (+) denotes sensitivity analyses performed by sequentially including specific covariates from the baseline model for the nine individual proteins (ANG, IL1R1, CXCL14, ESAM, PTGDS, HAVCR1, FGFR2, IGSF8, CCL3):Model 3: Baseline model fully adjusted for clinical covariates including age, sex, education, Townsend index, HbA1c, glucose, lipid measures, urea, BMI, eGFR, ACR, blood pressure, waist circumference.Model 3 + duration (green triangles): Sensitivity analysis based on Model 3 with the inclusion of diabetes disease duration.Model 3 + duration/comorbidities (yellow squares): Sensitivity analysis based on Model 3 with the simultaneous inclusion of both diabetes disease duration and baseline comorbidities (coronary heart disease, hypertension, dyslipidemia, and heart failure).Model 3 + duration/comorbidities/medications (red diamonds): Sensitivity analysis based on Model 3 with the simultaneous inclusion of diabetes disease duration, baseline comorbidities, and baseline medication use (antihypertensive drugs, lipid-lowering drugs, and insulin).Estimates are pooled across 10 multiple imputations (with 20 terations each). The vertical dashed line indicates the reference value of HR = 1.00.

**Supplementary Figure 6** Web Application Deployment

(https://jiangli2941.github.io/MACE-prediction-v2/). The interface enables clinicians to input Olink-derived NPX values for the 9 serum proteins and 19 clinical indicators, with real-time calculation of MACE outcome risk probability. A prominent disclaimer clarifies that the model was trained on NPX-scale data and that predictions are validated only for Olink Explore 3072 outputs, not for absolute concentrations from other platforms.
